# Curated Collections for Educators: Five Key Papers about Receiving Feedback in Medical Education

**DOI:** 10.7759/cureus.5728

**Published:** 2019-09-23

**Authors:** Sreeja Natesan, Christine Stehman, Rebecca Shaw, David Story, Sara M Krzyzaniak, Michael Gottlieb

**Affiliations:** 1 Emergency Medicine, Duke University Medical Center, Durham, USA; 2 Emergency Medicine, Indiana University School of Medicine, Indianapolis, USA; 3 Emergency Medicine, Gold Coast University Hospital, Queensland, AUS; 4 Emergency Medicine, Wake Forest Baptist Medical Center, Winston-Salem, USA; 5 Emergency Medicine, University of Illinois College of Medicine, Peoria, USA; 6 Emergency Medicine, Rush University Medical Center, Chicago, USA

**Keywords:** modified delphi method, curated collection, feedback, receiving feedback, formative assessment, medical education, post-graduate medical education

## Abstract

Introduction

Feedback is a complex, multi-component interaction that is essential for academic development and advancement. Successful feedback requires active involvement from both the giver and receiver. However, research and guidance on the subject mostly center on the role of the provider of feedback. But the receiver of feedback holds the true power in this interaction, choosing how to interpret the information and deciding whether or not to incorporate the feedback to instill behavioral change. In this article, the authors aim to summarize five key papers related to receiving feedback, in order to outline both relevant information for emerging clinician-educators and discern ways to use this information for faculty development.

Methods

In order to generate a list of key papers that describe the importance of receiving feedback, the authors conducted a consensus-building process informed by social media sources. Key articles on receiving feedback were aggregated through a literature search. This list was further augmented via an open call on Twitter for important papers regarding receiving feedback. Through these processes, a list of 43 papers was created on the topic of receiving feedback in medical education. After compiling this preliminary list, the authorship group engaged in a modified Delphi approach to build consensus on selecting papers that best described the process of receiving feedback.

Results

We present the group’s five most highly rated papers on the topic of receiving feedback in medical education. These papers were deemed essential and have also been summarized based on their relevance to junior faculty members and faculty developers.

Conclusion

While giving and receiving feedback are both vital for growth and development, much of the research focuses solely on giving feedback. However, receiving feedback is equally, if not more, important for instilling change in the learner. We explore the power of receiving feedback in medical education through five key papers that analyze the subject. We believe these papers can serve as great learning resources for both junior faculty members and faculty developers. They can assist the junior faculty to cultivate the ability to receive feedback and also serve as resources to aid senior faculty in building faculty-development sessions.

## Introduction

Feedback is an extremely complex process. It involves a multi-component interaction to convey information that is essential for personal and professional development. The intent of feedback is to improve knowledge and performance towards a common goal. A successful feedback exchange/interaction requires the active involvement of both the giver and receiver of feedback [1-5]. The giver of feedback must provide specific, timely information regarding an observed performance based on a benchmark. The receiver of feedback must decipher this information and decide whether to accept and incorporate it or not. As one might expect, any feedback interaction is riddled with challenges. Learning and, ultimately, overall success require both the giving of quality feedback as well as the incorporation of that feedback leading to behavior modification on the part of the receiver [6]. 

The available resources for feedback guidance often center on the role of the giver of feedback. There are plenty of articles and books that provide tips on how to become effective at the skill of providing feedback [7]. Typically, those who are involved in giving feedback are not aware of the intricacies and subtleties that those who receive feedback must deal with. However, the receiver of feedback holds the true power to interpret the information and make a decision to (or not to) incorporate the input received via feedback to instill behavioral change [8]. Usually, little effort goes into providing a clear and thorough understanding of giving and receiving feedback to clinicians during their training period, resulting in a poor feedback interaction for both the learners and faculty [1,3-5]. In this article, the authors aim to summarize five key papers on receiving feedback to outline relevant information for emerging clinician-educators and provide senior faculty with practical ways to use these resources for future faculty development. 

## Materials and methods

**Figure 1:**
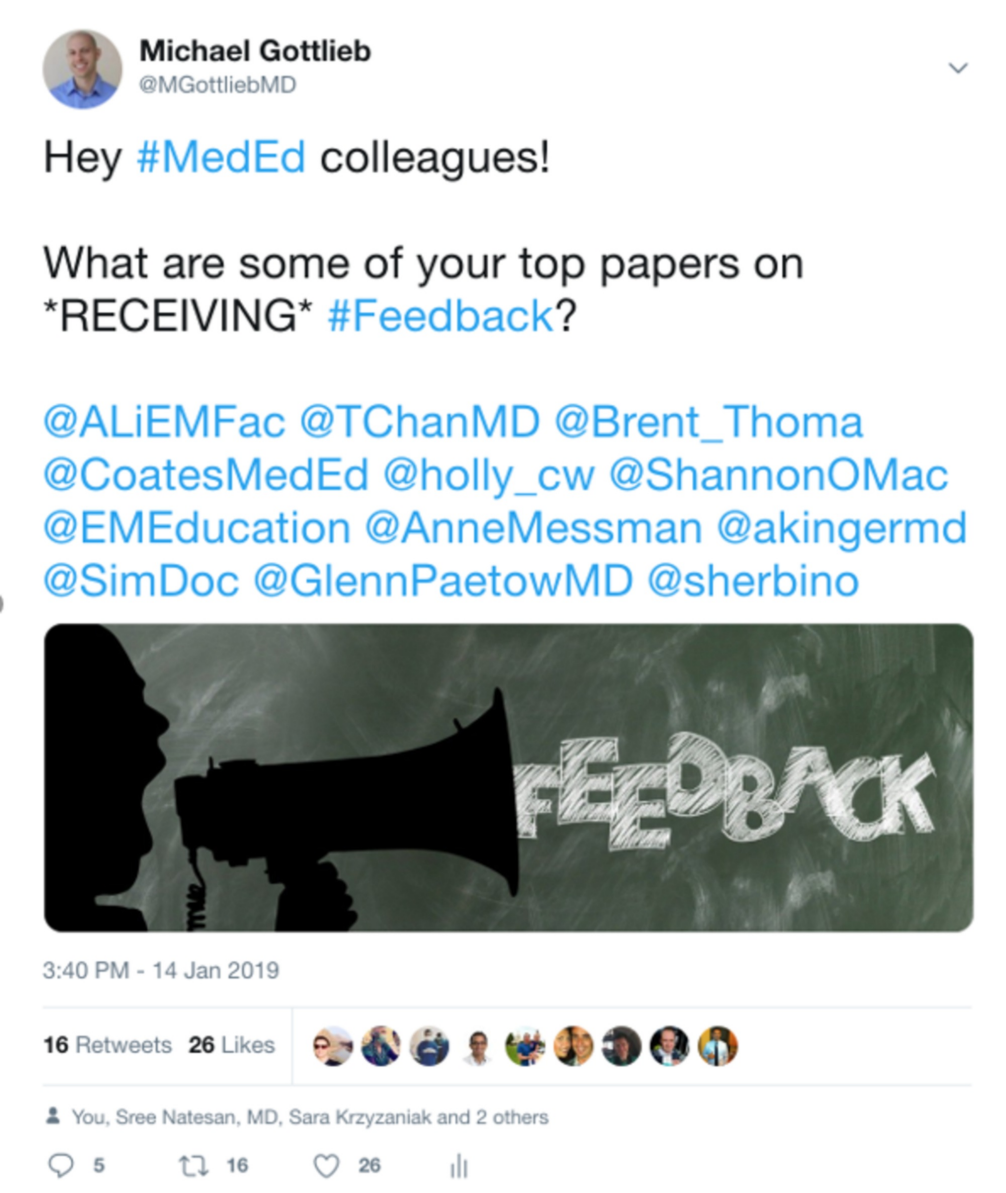
Twitter call for feedback articles

The article’s authors are all active members of the Academic Life in Emergency Medicine (ALiEM) Faculty Incubator program. The ALiEM Faculty Incubator program consists of an online community of 30 junior faculty members and 20 mentors who strive toward furthering medical knowledge for clinician-educators and enhancing opportunities for scholarships [9]. The first step in the process was to generate a list of key papers that describe the importance and significance of receiving feedback. To do so, the authors performed a literature search and compiled an aggregated list of relevant articles on receiving feedback. The authors searched PubMed and GoogleScholar to find articles relating to receiving feedback. Search terms included “feedback” and “medical education". This list of papers was further augmented via social media through an open call on Twitter (Twitter, Inc., San Francisco, CA) (Figure 1), requesting participants of the Free Open Access Medical education (#FOAMed) and general medical education (#MedEd) online communities to provide suggestions on important papers on the topic of receiving feedback [10]. The authors also reviewed the bibliographies of relevant articles for additional papers. Several papers in the list were suggested through more than one modality and, ultimately, these processes helped to create a list of 43 papers on the topic of receiving feedback in medical education. 

Because our consensus group included novices as well as experienced medical educators, the traditional Delphi methodology was not used [11,12]. Junior faculty, as well as experts in medical education, were invited to review the selected papers to ensure that they would be beneficial to a wide variety of educators throughout the span of their careers. Both were included based on the conviction that the process of feedback is not exclusive to experts, and junior faculty perspectives would bring value to the article-selection process. Novices were defined as junior faculty members and participants in the ALiEM Faculty Incubator. Experienced medical educators consisted of established clinician-educators who serve as mentors and facilitators of the ALiEM Faculty Incubator and have published >10 peer-reviewed articles.

As in previous articles in the Curated Collections for Educator series [13-23], the modified Delphi methodology was utilized. All 43 articles were read by all of the authors. The three-round voting method was used to evaluate the relevance of the papers to the process. Round one utilized a seven-point Likert scale with the statement "unimportant for junior faculty" placed at point one and "essential for junior faculty" at seven. The authors were asked to indicate the importance of each article based on this scale. The second round aimed to create a broad inclusion criteria for articles the authors deemed appropriate to be included as the “must be included” papers. With no limit to the number of articles that could be chosen, the authors were simply asked to indicate if each article "must be included in the top five papers" or "should not be included in the top five papers.” The results of the first round, including a frequency histogram displaying how each article had been rated, were provided. In the third and final round, the authors were given the results of the second round in the form of a percentage of raters who indicated that each article must be included. They were then asked to select five final, key papers that should be included in the article. 

## Results

Of the 43 papers compiled at the starting point of the modified Delphi process, a consensus on selecting five key papers that were most relevant to both the junior faculty and faculty developers was obtained. These were deemed to be the most essential for junior faculty on the topic of receiving feedback in medical education. Additionally, these papers were determined to be of interest and relevance to faculty-development course creators.

## Discussion

Each of the five key articles is summarized below. The commentaries discuss these papers with respect to their relevance to junior faculty, as well as importance in general medical education for future faculty development.

1. Algiraigri A: Ten Tips for Receiving Feedback Effectively in Clinical Practice [6]

Summary: the article focuses on the strategies to improve feedback receptivity using a comprehensive review of the literature as its basis. After discussing the limitations and strategies to improve self-assessment, the author provides 10 practical tips on receiving feedback. These strategies are presented in bullet points to allow for ease of reading and comprehension. The author also emphasizes the need for learners to connect with potential feedback providers and actively seek out feedback. Finally, the article discusses the importance of obtaining specific, tangible feedback and developing an action plan to incorporate the feedback into future endeavors.

Relevance to junior faculty members: faculty may perceive their primary role in the feedback interactions as the provider of feedback. While giving feedback, faculty must also strive to understand how feedback is received. This paper provides junior faculty with strategies to accept and integrate feedback from both learners and more experienced clinicians while offering insights on how they may set up the feedback interaction to maximize the impact on their learners. Additionally, junior faculty can use this to teach learners how to improve their feedback receptivity. Moreover, by understanding the skills to improve feedback receptivity, junior faculty can also identify strategies to facilitate effective delivery of feedback to others. Overall, this article includes practical tips that are easy to read and incorporate into practice. 

Considerations for faculty developers: faculty developers can utilize this review to help design sessions to improve feedback receptivity among other faculty. Additionally, the table in the manuscript provides a summary that could be provided to the faculty as a guide on how to improve one’s feedback receptivity. Finally, this article can also be a useful tool for faculty developers when receiving feedback as it pertains to their own faculty development courses and other initiatives.

2. Davies K And Guckian J: How to Ask for and Act on Feedback: Practical Tips for Medical Students [24] 

Summary: Davies and Guckian begin this article by discussing the need and importance of feedback within medical education. Based on the purpose of feedback on closing the gap between actual and desired performance, the authors acknowledge the challenge of requesting feedback in the setting of clinical practice. Tips for overcoming this challenge are then outlined. Overall, the authors emphasize that the learner is the driving force for successful feedback interactions. Specifically, self-assessment, specific goal-setting, and clarification are learner-driven components of feedback. Feedback should be dialogical and should be sought by multiple sources including educators, peers, and patients. After discussing the learner-centered nature of the feedback interaction, the article moves on to the topic of evaluating and integrating the feedback received. In particular, it discusses how the lens through which the feedback process is viewed impacts the learner’s attitude toward the advice. Feedback should be perceived as an opportunity for improvement and should be based on actions, not personality or character. Isolating the key points for later reflection permits comparison to the learner’s self-assessment. As goals are reached, learners should generate new objectives with the goal of continuous performance improvement. Finally, the authors discuss how solely negative feedback impacts learners and the value of reinforcement of positive actions.

Relevance to junior faculty members: although this article was written primarily for medical students, many of the tips discussed can also be utilized by faculty members. Because residency training does not typically focus on giving and receiving feedback, this article can serve as a framework to highlight the complexities of this interaction. Specifically, it can teach faculty physicians to inquire about self-assessment and goals from the learner prior to a feedback session. To ensure that what is being given and received is the same, the educators can then ask the learner to explain their understanding of the feedback provided. The open-dialogue component can serve as a framework to allow both sides to practice giving and receiving feedback, which is crucial to a successful exchange. 

Considerations for faculty developers: while learners should be actively seeking out feedback to facilitate clinical improvement, the barriers to this happening regularly or effectively should be acknowledged. Educators should be equipped to bridge this potential gap in order to provide feedback to students. Faculty developers can utilize the tips proposed by Davies and Guckian to instruct teaching physicians on how to create specific opportunities for feedback provision by eliciting self-assessments and inquiring about a learner’s goals. Additionally, having some tools to evaluate why a student is not incorporating feedback may provide a blueprint for changing the receptivity of the learner. Faculty developers may also choose to use these tips as a basis for faculty reflection on their recent feedback interactions, both as givers and receivers of feedback. 

3. Kowalski K: Giving and Receiving Feedback: Part II [25]

Summary: Kowalski begins with a description of feedback and the benefits associated with receiving feedback well. The article then goes on to discuss different strategies and tips that can be fostered to support a constructive approach to receiving feedback. One such conceptual approach, the ACT model (Accept, Clarify, and Thanks) developed by Baldoni is explained in brief, followed by further useful tips to support receiving feedback. Some examples of these tips include suspending judgment until after the meeting and asking the person to suggest alternative behaviors. The article then goes on to describe behaviors that are not helpful when receiving feedback and concludes by delving into the value of reflection after feedback conversations.

Relevance to junior faculty members: the ability to receive feedback and utilize it well is an important but often under-taught skill for faculty, residents, and students. This article provides a useful summary of tips to help learners engage in useful feedback conversations and make the most of the feedback they receive. Junior faculty can also use these tips to refine their skills in receiving feedback on their own performance.

Considerations for faculty developers: this brief article can provide a valuable starting point for faculty developers wishing to provide junior faculty with an overview of the importance of receiving feedback. The article highlights tools that can be used, such as the ACT model, to improve their own teaching and clinical practice. Faculty developers could draw on the practical tips provided in the article to help structure faculty development sessions on receiving feedback. They can recommend this as a pre-reading assignment prior to a feedback-receiving session.

4. Ramani S, Könings KD, Ginsburg S, Van Der Vleuten CPM: Twelve Tips to Promote a Feedback Culture with a Growth Mindset: Swinging the Feedback Pendulum from Recipes to Relationships [26]

Summary: the likelihood that a learner will accept and assimilate feedback is closely linked to a positive feedback culture that promotes a growth mindset (i.e., the belief that success results from hard work and failures lead to learning) [27]. A feedback culture is a complex, poorly defined concept that is impacted by the learner, instructor, and contextual considerations. This article uses existing literature to define the various influences on feedback culture within several domains: feedback provider, feedback recipient, feedback relationship, and institutional context. The result is 12 practical strategies that can be implemented to strengthen the feedback culture, encourage a growth mindset among learners, and provide feedback that is aimed at professional development. A few examples of the strategies include promoting a positive learning environment, using direct observations for feedback, and facilitating informed reflection and self-assessment.

Relevance to junior faculty members: a primary goal for junior faculty wishing to improve their feedback giving is to establish credibility with their learners as this is linked to higher acceptance of feedback. The tips discussed in this paper include strategies to help create a positive learning climate. In addition, faculty are encouraged to perform frequent direct observations of their learners as this improves feedback credibility. Junior faculty should also help their learners through a guided self-assessment and reflection on their own strengths and weaknesses, as feedback that is incongruent with a learner’s self-image is often rejected. The ability to nurture a growth mindset in learners is critical to helping them assimilate feedback, particularly when faculty utilize performance-directed language and nurture a formative assessment setting that balances ego costs (resulting from constructive feedback) and ego benefits (resulting from reinforcing feedback).

Considerations for faculty developers: faculty developers’ role in improving a learner’s reception of feedback is two-fold. Primarily, faculty developers often find themselves in a position to shape institutional influences that directly impact the development of a positive feedback culture. One goal should be to normalize constructive feedback at all levels of the profession and set an expectation for ongoing formative feedback. This can be accomplished through training teachers and learners “in the use of language framed in a continuous practice improvement approach.” Secondly, professional development should be offered to learners and faculty in receiving and assimilating feedback. These sessions should include background on an approach to training that utilizes a coaching structure as well as frameworks to promote positive faculty-learner relationships and delivery of feedback, such as the “educational alliance”, the Johari window, the Plus Delta feedback approach, and the participatory design loop for feedback conversations.

5. Ten Cate OT: Why Receiving Feedback Collides with Self Determination​​​​​​​ [28]

Summary: Ten Cate uses this article to discuss self-determination theory (SDT) and how the components of the theory explain why learners often do not use the feedback given to them. The author starts by reminding readers that while feedback is essential for learner development, and many instructors believe they do an effective job of providing feedback, learners often do not receive and act on that feedback. The author then introduces Deci and Ryan’s SDT, which suggests that motivation is related to feelings of competence, autonomy, and relatedness. The author then discusses how critical but constructive feedback could conflict with each of these domains, suggesting that these conflicts are the reasons why learners do not receive and incorporate feedback. First, feedback may be received as indicating the learner is not as competent as they would like and believe. Second, the learner often has no say in when and how he receives feedback. Third, the emotions associated with the feedback may affect the relationship the learner has with the person providing the feedback or the other way around. Finally, the author discusses how to give feedback in ways that do not conflict with the domains of SDT in order for the learners to be motivated to incorporate the feedback into their development.

Relevance to junior faculty members: while this article specifically addresses how to give feedback in ways that do not conflict with SDT, anyone receiving feedback could use the information provided as well. Receiving feedback, especially negative feedback, is difficult and acting on that feedback is often more difficult. If those receiving feedback can understand the domains of SDT and how feedback might conflict with those domains, they can take steps to mitigate those conflicts. Taking the time to work with those who provide feedback to establish times, locations, and methods that work well for both parties may help to improve the feelings of autonomy. Establishing relationships is crucial to ensure that the receiver of feedback understands that the feedback is given from a place of caring. 

Additionally, making sure to provide feedback, especially negative feedback, in a private setting and not comparing faculty may mitigate any feelings of being the “only one” or one on the outside. Finally, after receiving negative feedback, faculty should take a minute and recognize that while this feedback may conflict with their feelings of competence, that does not mean that the feedback provided does not contain any important data. Rather than just ignoring the feedback, faculty should take a figurative step back, recognize and accept the emotions that come with hearing something negative, and then critically and calmly evaluate the information that has been provided with an eye towards how it could be incorporated. In addition, junior faculty may choose to use these domains to work with their learners to reflect on their perceptions of these domains and how those perceptions may influence a feedback interaction.

Considerations for faculty developers: faculty developers could use the information provided in this article in two ways. First, they can educate faculty on SDT so that faculty can be aware of the effects it might have on how learners receive feedback. As part of this education, faculty could be encouraged to develop relationships with learners that establish mutual trust and understanding in order to increase relatedness, while also considering starting any feedback conversation with a nod to the learners' autonomy: “I would like to provide you with some feedback. How would you prefer to receive it (e.g., written, verbal), and when would be a good time for us to discuss it?”. The other way faculty developers could use this paper is to work with faculty on how they receive and incorporate feedback, discussing the process mentioned above of stepping back to remove the emotion and reflect on the information rather than just rejecting negative feedback outright. If done well, faculty should be able to model this behavior for learners. 

Limitations

A curated collection for educators differs in its methodology with other research articles such as a systematic review. As a result, the search strategy and rigor were not designed to be exhaustive. Although this was done on purpose, it is possible that some relevant articles may have been missed. However, consultation with medical education experts, in combination with an open social media call, did help to bridge gaps and allowed the authors to identify a list of the most important articles on receiving feedback. We also reviewed references of the papers obtained to determine any missing articles that would be pertinent. Another potential limitation was the use of the modified Delphi instead of the traditional expert-only Delphi. We believe both experienced and junior faculty hold expertise in recognizing tools pertinent to the subject under discussion, while a pure Delphi approach involves only topic experts.

## Conclusions

While giving and receiving feedback is vital for growth and development, much of the research on the subject focuses solely on giving feedback. In this review, the significance of receiving feedback is explored through five key papers for receiving feedback in medical education. These papers provide tips and frameworks that can serve to help junior faculty grow in their ability to receive feedback and also serve as resources on which senior faculty can build faculty-development sessions. These articles are applicable to physicians at all stages in their careers and can help them to promote a healthy feedback environment in medical-education setting.
